# Editorial: Developing combined modality therapy with mitochondria-targeting strategy

**DOI:** 10.3389/fonc.2022.1111724

**Published:** 2023-01-06

**Authors:** Yong Teng

**Affiliations:** ^1^ Department of Hematology and Medical Oncology, Winship Cancer Institute, Emory University, School of Medicine, Atlanta, GA, United States; ^2^ Wallace H. Coulter Department of Biomedical Engineering, Georgia Institute of Technology & Emory University, Atlanta, GA, United States

**Keywords:** mitochondria and disease, targeting strategy, combined therapy, translational sciences, cancer

A broad array of molecular and cellular events is associated with developing resistance to treatment, such as deregulation of the cell cycle, inhibition of DNA damage repair mechanisms, and metabolic alterations. Most cancer therapy-induced responses, including resistance, involve the dysfunction of mitochondria. Mitochondria have acquired numerous functions throughout evolution, controlling energy production, cellular metabolism, cell survival, apoptosis and autophagy within host cells. Tumor cells can develop defects in mitochondrial function, presenting a potential strategy for designing selective anticancer therapies. However, non-specific targeting of mitochondrial functions may have significant unwarranted effects on normal cell growth and survival. Therefore, treatments conjugated with other anticancer therapy are needed to precisely target specific mitochondrial proteins involved in tumor progression and the acquisition of treatment resistance.

In the present Research Topic, we have garnered several contributors to provide evidence‐informed insights into the mechanistic and pathogenic role of mitochondrial proteins to support the discovery of novel therapeutic targets for directed mitochondrial treatment and eventually facilitating enablers of knowledge translation. After a rigorous peer-review process, seven articles have been collected, consisting of two comprehensive review and five original research articles. Notably, these articles were contributed by renowned academic institutes from North America, Europe and Asia engaged in mitochondria-based biology and translational research, demonstrating the great interest in this hot area.

Cancer cells exhibit metabolic plasticity that endows them with a selective advantage to face harsh microenvironmental alterations and orchestrate nutrient sensing and upload, signaling, and redox circuits. The finely tuned reactive oxygen species (ROS) generation and scavenging within a certain sub-toxic tumorigenic range are two aspects fundamental to cancer cells as ROS mediate cell signaling to significantly impact a wide range of pathways involved in cancer development and progression. That is the reason why ROS have recently become an attractive target for anticancer therapies. Ippolito et al. outlined several crucial functions of mitochondrial redox activity in different cancer stages by elaborating effects of mitochondrial ROS (mROS) on tumor initiation, progression, energy metabolism, stemness achievement, metastases and tumor immune environment. They also explored the impact of mROS on treatment response in cancer, such as tumor chemosensitivity. Because mitochondrial redox homeostasis crucially regulates cancer cell behavior and several cancer hallmarks, the authors also attempted to outline the potential redox-based targeted therapeutic strategies, either single or combined modality. It is worth mentioning that two new mROS-targeted treatments, photothermal and photodynamic therapy, have rendered their effectiveness in curing and preventing cancer. Criscuolo et al. reviewed the available literature on the coordinated regulation of mitochondrial and cytosolic mRNA translation, as well as their effects on the integrity of the mitochondrial proteome and functions. The purpose of this review paper is to highlight the importance of mitochondrial protein quality control systems in coordinating mitochondrial and cytosolic protein translation. More than that, this critical review also hints that mitochondrial protein homeostasis dysfunctions are tightly associated with cancer development, and thus the most relevant therapeutics hold great promises as anticancer strategies.

Mitochondrial dependency of leukemia cells and their altered oxidative metabolism have already been explored as a common abnormality existing in acute myeloid leukemia (AML). Accumulating evidence suggests that AML may be particularly sensitive to chemotherapeutics that target mitochondria. To further investigate this sensitivity, a research group at Rice University screened 36 drug combinations randomly paired one of six mitochondria-targeting compounds (IACS-010759, rotenone, cytarabine, etoposide, ABT-199, and carbonyl cyanide m-chlorophenylhydrazone) with one of six compounds with other activities (midostaurin, dasatinib, 2-deoxy-D-glucose, 3-bromopyruvate, lonidamine, and vinorelbine) in two AML cell lines (Panina et al.). Among these drug pairs, IACS-010759/vinorelbine impaired ATP level and disrupted mitochondrial respiration and coupling efficiency most profoundly, which also retained high synergy and strong selectivity in primary AML cells. This study supports the potential of mitochondria-based drug cocktails for future therapeutic development and optimization and reinforces the value of this approach.

Interestingly, by performing genome-wide CRISPR/Cas9 library knockdown screening, a Chinese research team identified that mitochondrial elongation factor 2 (MIEF2) was among the top candidate genes in oxaliplatin (OXL) resistant cell lines (Xie et al.). Mechanistically, downregulation of MIEF2 impaired mitochondrial stability and suppressed cytochrome C-dependent apoptosis, and eventually enhanced colorectal cancer cell resistance to OXL. These novel findings suggest that MIEF2 represents a predictive biomarker of OXL responsiveness and targeting it could improve chemotherapeutic effectiveness in colorectal cancer. In the same type of cancer, TRIAP1, a chaperone interacting with the Ups/PRELI family proteins, participates in the intramitochondrial transfer of lipids to synthesize cardiolipin and phosphatidylethanolamine. Nedara et al. reported that depletion of TRIAP1 induced extramitochondrial perturbations, leading to remarkable changes in the endoplasmic reticulum-dependent lipid homeostasis and induction of a p53-mediated stress response. This depletion also conferred strong p53-dependent resistance to the metabolic stress mediated by glutamine deprivation. Another original research collected in this Research Topic shows that mitochondrial morphology and dynamics are altered during ovarian cancer progression and upon aggregation (Grieco et al.). In this study, the researchers utilized the mouse ovarian surface epithelial (MOSE) model for serous ovarian cancer and analyzed the morphological and functional changes that occur during the progression from benign (MOSE-E), to slow (MOSE-L) and fast-developing disease (MOSE-L_TICv_), and in response to aggregation and hypoxia. The resulting findings gain new insight into how the alterations in mitochondrial morphology contribute to the survival of aggregates in the non-permissive environment of the peritoneal cavity during metastasis.

Mitochondria are the powerhouse of the cell, while the liver is the center of human metabolism at the whole organism. Thus, identifying mitochondria localized proteins as prognostic biomarkers for hepatocellular carcinoma (HCC) will be interesting. Zhang et al. analyzed HCC cohort database in The Cancer Genome Atlas (TCGA) and constructed a classifier containing 10 mitochondrial-related genes (Mito-RGs) for predicting the prognosis of HCC. The results from bioinformatics reveal cross-talk between metabolic processes governing bile acid and the infiltration of tumors by immune cells, providing an innovative strategy for HCC metabolic therapy based on the modulation of mitochondrial function.

Although mitochondrial oncology has been known for much longer than 20 years, the field has experienced a resurgence of interest in the past decade. We understand that a large proportion of this journal’s readers are basic and clinical cancer researchers and as such wanted to give them a bigger voice in the journal’s content. Beyond cancer, mitochondrial defects are often the underlying cause of other diseases. The articles within our collection add new value to the development of mechanism-informed therapeutic strategies that place the organelle as a promising target.

## Author contributions

The author confirms being the sole contributor of this work and has approved it for publication.

